# Associations between parenting partners' objectively-assessed physical activity and Body Mass Index: A cross-sectional study

**DOI:** 10.1016/j.pmedr.2015.06.007

**Published:** 2015-06-11

**Authors:** Jesmond Zahra, Russell Jago, Simon J. Sebire

**Affiliations:** Centre for Exercise, Nutrition & Health Sciences, School for Policy Studies, University of Bristol, 8 Priory Road, Bristol, BS8 1TZ, UK

**Keywords:** Accelerometery, Physical activity, Obesity, Body weight, Health behaviour

## Abstract

**Objective:**

Family members have the capacity to influence each other's health behaviours. This study examined whether there were associations in the objectively assessed physical activity and Body Mass Index (BMI) of mothers and fathers.

**Methods:**

Recruitment took place in Bristol (UK) during 2012/13. Participants were 272 pairs of parents (dyads) that wore an accelerometer for at least 500 min on 3 or more days. Parents provided demographic information and self-reported height and weight. Multi-variable linear and logistic regression models examined the relationships between parents' moderate-to-vigorous physical activity (MVPA) and BMI.

**Results:**

MVPA minutes (r = 0.26, p < 0.001) and Body Mass Index (r = 0.20, p = 0.002) of parents were correlated. Logistic regression analysis showed that mothers were almost twice (OR 1.87, p < 0.05) as likely to be overweight or obese when fathers were. Linear regression models showed that at the weekend every 9 min of paternal MVPA was associated with 3 min of maternal MVPA (r = 0.34, p < 0.001).

**Conclusions:**

Both physical activity and BMI of parenting partners were associated. Since parents tend to share home environments and often perform activities together or as a family, then behavioural changes in one parent may have a ripple effect for other family members.

## Highlights

• Accelerometer derived levels of MVPA in parents are associated.
• Every 9 min of father MVPA predicts a 3 minute increase for mothers.
• Self-reported BMI scores of mothers and fathers are associated.
• Parents accumulated approximately 10 min less MVPA at the weekend.
• Behaviour change in one parent may have a knock on effect for a spouse.

## Introduction

Regular physical activity (PA) has both preventive and rehabilitative effects for many chronic health conditions (Bauman, 2004, Donaldson, 2004, KOKKINOS and MYERS, 2010). Approximately 33% of men and 45% of women in the UK fail to meet the recommended 30 min of moderate-to-vigorous physical activity (MVPA) on 5 days per week (Health and Social Care Information Centre, 2012). Furthermore, it is estimated that 66% of men and 57% of women (aged ≥ 20) within the UK are overweight or obese (Ng et al., 2014). Low levels of PA have been associated with overweight and obesity in adults and children and recent data suggests that individuals who meet the PA guidelines are at reduced risk of being overweight or obese (CHAU et al., 2012, HILLS et al., 2011, Health & Social Care Information Centre, 2012). There is a need to develop strategies to increase PA and reduce obesity. Designing successful interventions requires an understanding of the determinants of PA behaviours; those interventions that target casual determinants of behaviour are likely to be more effective (Michie et al., 2008).

Families and family environments are proposed to be central components in both adult and child PA behaviours and choices. Diet and physical activity are both influenced by core relationships within the home (Wilson, 2002) and therefore spouses are likely to be influential when it comes to modifying and maintaining healthy behaviours. Studies examining spousal correlations in PA levels have provided evidence of a relationship. A pedometer study conducted in Northern France identified an inter-spousal correlation during weekend days (r = 0.14); there was no evidence of an association during week days (Jacobi et al., 2011). These findings are consistent with the analysis from the Quebec Family study which highlighted an inter-spousal PA relationship (Simonen et al., 2002). This was the strongest for self-reported past year PA data (r = 0.43). Further analysis of previous three day PA data, recorded via diaries, demonstrated a correlation between spousal moderate-to-strenuous activity (r = 0.22). However, measuring PA using self-report can result in measurement error, bringing into question the validity of the findings and the accuracy of the reported correlations (STERNFELD et al., 2012, PRINCE et al., 2008). Previous studies have been limited by the use of pedometer or self-reported collected PA data; neither of which allows for the calculation of important output measures, such as minutes in MVPA. Further research into spousal PA association is required that utilises objective measures in order to provide a more reliable indication of spousal PA associations.

Research has demonstrated that health-related behaviours of spouses converge over time (Meyler et al., 2007). It is therefore possible that spousal body composition may be similar between parents. Both marriage and parenthood are associated with decreased PA and increased body weight in males and females (HULL et al., 2010, AVERETT et al., 2008) suggesting that individuals' behaviours may become similar once in a relationship. Previous research has demonstrated that changes from an unhealthy to a healthy behaviour in one spouse are associated with a positive change by the other partner (Falba and Sindelar, 2008). In addition, there are some evidences of associations in spousal Body Mass Index (BMI) (JEFFERY and RICK, 2002, Abrevaya and Tang, 2011, JACOBI et al., 2011). Within the UK there are very few studies that have explored spousal BMI and even fewer with a specific focus on parents. The current study examined the spousal associations in parental PA and BMI among parents with children aged 5–6 years. Through utilising an objective measure of physical activity we hope to build on the findings of previous spousal PA studies. The main aim of the study is to investigate if the BMI and physical activity (MVPA) of parent dyads are associated.

## Methods

### Study design

Data are from a cross-sectional study (B-ProAct1v) carried out at the University of Bristol. The study aimed to examine factors that influenced young children's (5–6 years) and parents' PA, with a specific focus on the influence of parents on child PA behaviours (JAGO et al., 2014b, JAGO et al., 2014a). Data were collected from 57 primary schools in the greater Bristol area between January 2012 and May 2013, during this time 250 primary schools in Bristol and the surrounding areas were invited to participate. Ethical approval was granted by the School for Policy Studies research ethics committee at the University of Bristol and written informed consent was obtained from parents.

### Participants

**Fig. 1:**
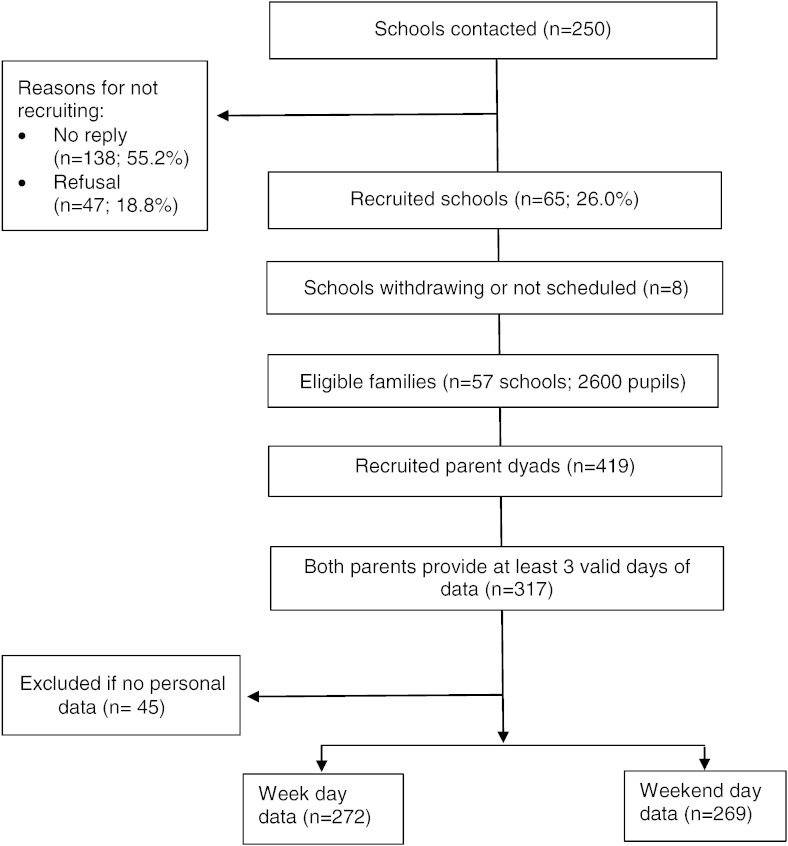
Study flow of participants. Based on UK participants — 2012/13.

To take part in the project children and at least one parent needed to participate; second parents were encouraged to take part but it was not a requirement of the project. In total 1456 pupil and parent dyads agreed to take part. 1267 child–parent dyads wore and returned an accelerometer and were included in the final dataset. For the current study we were interested in parent dyads (e.g., mother–father) and therefore only cases where both parents participated are included in our analysis. Fig. 1 shows the study flow of participants.

### Measures

Participating parents (first parents) were required to complete a questionnaire about family characteristics, personal demographics, overall child health and questions relating to screen-viewing behaviours. Second parents were asked to report demographic information. All parents that took part were ask to report their gender, height and weight to enable the calculation of BMI (BMI = kg/m^2^). In addition, parents (first and second) and children wore an Actigraph GT3X accelerometer for five days; three week days and two weekend days. Uniaxial data were processed using Kinesoft (v3.3.75; Kinesoft, Saskatchewan, Canada) software which estimated minutes of MVPA for parents based on an age-appropriate cut point (≥ 2020 counts per minute) (Tudor-Locke et al., 2010). Sedentary behaviour and light activity were omitted from the analysis as they cannot be analysed in relation to UK PA government guidelines. Mean counts per minute (CPM) were also calculated. This measure provides an indication of the volume of physical activity in which participants engaged.

A valid day was specified as ≥ 500 min of wear time, this was to prevent further reductions in the dataset and to conform with the method used in previous studies working with the same data (Jago et al., 2014a). Any period of 60 min or more of consecutive zero counts, with an allowance of 2 min of interruption, was classified as non-wear time and removed from subsequent analysis. Participants' home address and postcode were reported and then linked to lower layer super output data enabling the calculation of Index of Multiple Deprivation (IMD) scores based on the English Indices of Deprivation (http://data.gov.uk/dataset/index-of-multiple-deprivation).

### Data management/reduction

In total 317 participating dyads met the inclusion criteria for at least three valid days of accelerometer data. Participants that did not provide personal demographic information were removed from the subsequent analysis. Following this criteria 272 participants were included in the final dataset, of which 269 provided at least one valid weekend day of wear time (wear time minutes, M = 798, SD = 66, Range = 579 to 949). Daily minutes of MVPA were calculated as a continuous variable. Participants were also categorised as either meeting or failing to meet the recommended 30 min/day (World Health Organization, 2010). Similarly BMI was computed as a continuous variable and also dichotomised to express weight status; normal weight or overweight/obese as defined by the UK National Health Service (http://www.nhs.uk/Conditions/ Obesity/Pages/Introduction.aspx).

### Statistics

Student t-tests were used to explore differences in demographic factors, BMI and MVPA between included and excluded participants. Descriptive statistics (i.e., means and standard deviation) were calculated and correlations were used to examine relationships between parents' MVPA and BMI. Linear regressions were conducted for each outcome variable and separately for weekday and weekend days to enable comparisons. Female data was used as the outcome and male data as the exposure. Logistic regression analysis was used to examine whether fathers meeting the recommend 30 min of daily MVPA was associated with mothers meeting the same guideline. Additional logistic regression analysis examined the odds of mothers being overweight/obese when fathers were above the BMI threshold for normal weight status. Each model was adjusted for female age, BMI, employment status, number of children, household cars and IMD scores as these have been previously associated with PA in adults (TROST et al., 2002, CERIN et al., 2009, HULL et al., 2010). Since participants were recruited via local primary schools, robust standard errors were used in all models to account for clustering of participants in schools. Analyses were conducted in Stata version 13.1 (Statacorp, College Station, TX). The R^2^ value has been added to the regression model to provide an indication of the variance in outcome variables explained by exposure variables in this sample (Pearce, 2011).

## Results

Descriptive statistics are shown in Table 1. On average males were approximately 1 year older than females and had slightly higher BMI. Males accumulated more MVPA minutes than females; fathers averaged 52 min of MVPA per day overall, and 56 min during weekdays. Both males and females performed approximately 10 min less MVPA on weekend days.

Parental BMI was positively correlated (r = 0.20 p < 0.001). Adjusted linear regression analysis showed that an increase of one unit in male BMI was associated with an increase of 0.22 units (p < .001) in mothers, which could be interpreted as an increase of one unit (kg/m^2^) per five unit increase in father BMI. Adjusted logistic regression analysis showed a mother was 87% more likely to be overweight or obese if the father was overweight or obese (Table 2).

There was strong evidence of an association between parental PA (r = 0.26, p < 0.001). Table 3 displays the results from linear regressions performed on MVPA data. Mothers' weekend activity increased by 0.34 of a minute per extra minute of father MVPA, or an extra 3 min for every 9 min of father MVPA. During week days the relationship between parental PA was weaker; the MVPA of mothers increased by approximately 8 s per minute of father MVPA.

Logistic regression models for parental PA are shown in Table 4. At weekends mothers were over 90% more likely to meet the recommended 30 min of MVPA if fathers did so in both unadjusted and adjusted models. There was no evidence of an association for weekdays.

## Discussion

The data presented in this paper shows strong evidence that both the PA and BMI of parents is associated. Associations between partners' PA were strongest during the weekend. Linear regression models demonstrated that an increase of 9 min of MVPA performed by fathers was associated with approximately three additional minutes in mothers. Weekend associations were evident even when PA data were dichotomised; mothers were 91% more likely to meet the recommended daily MVPA when the father did.

To the best of our knowledge the current study is the first to use accelerometery to examine associations between parents' physical activity levels. Other studies using different methods have reported similar findings, but the inter-spouse PA associations reported have tended to vary significantly between studies (SIMONEN et al., 2002, MAIA et al., 2013, JACOBI et al., 2011). This variation may be due to the variety of measures used to record PA. Our results provide strong evidence of a relationship between parents' MVPA and our analysis provides further evidence that parents influence each other's health-related behaviours. Weekday parental MVPA was only weakly associated which could be a result of the parents' working patterns, different commuting modes, the amount of PA accumulated through employment or having fewer opportunities to be active together on weekdays. Of the dyads included in the analysis over 98% reported that at least one parent works, therefore during the working week it is probable that behaviours are more independent and spousal influence on PA behaviour is less predominant.

BMI was associated between parents, with regression models indicating an increase of one unit for mothers per additional five units of male BMI. The association was also present when using dichotomised variables in logistic regression models. This is in line with previous findings that have demonstrated that the BMI of spouses is associated (CHEN et al., 2014, BROWN et al., 2014). Evidence from a longitudinal study that tracked the BMI of married couples for two years indicates that changes in weight-related behaviours, such as dieting or exercise may be influenced by spousal behaviour (Jeffery and Rick, 2002). Since parents tend to cohabit it is likely that their eating behaviours and diets are similar, which could partly explain the results.

Although the current study demonstrates associations between spouses, due to the cross-sectional nature we are unable to determine the pathways or mechanisms driving these behaviours. However, it is probable that there are a number of plausible explanations. Firstly, BMI associations could be a function of shared cues, routines and opportunity for eating within the home. Furthermore, parents may share similar preferences for specific foods, thereby increasing or reinforcing each other's consumption of these foods. Similarly, PA levels are likely to be influenced by preferences for type and volume of activity. With this in mind individuals living with an active partner may be more motivated and encouraged by their partner to become more active. Additionally, spousal support, attitudes and lifestyle choices of spouses may play a substantial role. If parenting partners place a similar value on the importance of being active, they may strive to ensure they participate in regular PA. It would be useful to explore these associations in a longitudinal study in order to develop a better understanding of the underlying mechanisms behind these associations.

The findings from this study raise a number of issues for researchers and others who are designing behavioural change interventions. If behavioural changes in the PA of one parent have a knock on or ripple effect, then it would be worth considering this throughout the development, data collection and evaluation processes of an intervention. Moreover, if parents can influence their children's and each other's health behaviours, interventions aiming to increase parents' PA should strive to encompass advice or guidelines on methods to encourage other family members to be active.

### Study strengths and limitations

The major strength of this study is the objective assessment of parental PA. Through using accelerometery it has been possible to obtain robust estimates of MVPA minutes of parents with young children. Furthermore, by collecting PA and BMI data from both parents we were able to examine individual parental data whilst also exploring associations between parental data. The collection of weekday and weekend MVPA data allowed for comparisons to be made. However, there are a number of limitations that should be considered. First, since we collected our data using accelerometers we are limited in our understanding of the types of activity in which parents engaged in and the extent to which associations reflect shared activity. Additionally, the use of questionnaires to collect demographic information limited our sample size. If one or both parents failed to complete the survey, they were excluded from the analysis since we could not control for potential confounding variables. This resulted in our sample size being reduced by approximately 15%. Furthermore, the collection of self-reported height and weight may have resulted in poor estimates of BMI, as has been demonstrated in previous studies (Gosse, 2014, NYHOLM et al., 2007). Although our analysis controlled for confounding variables it would have been beneficial to collect and adjust for other individual factors known to correlate with PA, such as self-efficacy or health status (Trost et al., 2002). To minimise parent burden, the questionnaire focused more on child than parent behaviour and health, thus we have no information on these potentially confounding variables. Finally, the cross-sectional design of the study prevents the assessment of the direction of the associations observed.

## Conclusion

This study has identified associations between parental BMI and PA levels. The use of an objective measure of PA further advances the knowledge about parental or spousal PA associations. Mothers are almost twice as likely to be overweight or obese when fathers are, suggesting that spousal behaviours and shared environments may result in parents sharing common risk factors for obesity. Analysis of accelerometer data revealed associations between parents' MVPA levels during week and weekend days. Stronger associations were identified at the weekend, which could suggest that families are more active together during this period.

## Funding source/trial registration

Data presented in this paper were funded by a British Heart Foundation project grant (ref PG/11/51/28986). JZ is funded by a British Heart Foundation studentship (FS/13/38/30319). The funder had no involvement in data analysis, data interpretation or writing of the paper.

## Conflict of Interest

The authors declare that there are no conflicts of interest

## Figures and Tables

**Table 1:** Demographic information including age, BMI and activity data. Based on UK participants - 2012/13.

		Fathers		Mothers				
N	Mean	SD	Mean	SD	Difference in means	95% CI	P
Age (years)	269	39.96	5.66	38.37	5.22	1.59	1.00 to 2.18	< .001
BMI (kg/m^2^)	269	25.86	3.64	24.81	4.23	1.05	0.45 to 1.65	< .001
CPM (overall)	272	408.15	145.14	397.03	129.58	11.12	− 8.37 to 30.60	.131
CPM (weekdays)	272	420.32	179.68	413.31	147.93	7.01	− 18.31 to 32.34	.293
CPM per weekend day	272	389.95	178.33	372.81	166.85	17.13	− 6.31 to 40.58	.076
MVPA minutes per day	272	51.64	22.08	48.12	21.34	3.53	0.37 to 6.69	.014
MVPA minutes per weekday	272	55.97	28.21	53.21	24.36	2.76	− 1.33 to 6.85	.097
MVPA minutes per weekend day	267	45.46	26.01	40.65	26.17	4.81	1.21 to 8.40	.004

BMI — Body Mass Index, CPM — counts per minute, MVPA — moderate-to-vigorous physical activity.

**Table 2:** Logistic regression analysis displaying odds ratios for females being overweight/obese, predicted by male overweight/obese group. Based on UK participants — 2012/13.

	OR	95% CI	p
Unadjusted	1.86	1.14 to 3.08	0.013
Adjusted^a^	1.87	1.13 to 3.09	0.014

a Adjusted for age of the mother, number of children, number of cars, employment and household IMD.

**Table 3:** Linear regression models for activity level data in which female PA is predicted by male PA. Based on UK participants — 2012/13.

	Unadjusted				Adjusted^a^			
Mean increase in mothers' PA (per minute) from one unit increase in fathers'	R^2^	95% CI	P	Mean increase in mothers' PA (per minute) from one unit increase in fathers'	R^2^	95% CI^a^	P
CPM overall	.266	.09	.13 to .41	< .001	.259	.13	.12 to .39	< .001
CPM weekdays	.142	.03	.01 to .27	.035	.132	.08	.01 to .26	.042
CPM weekend	.342	.13	.24 to .47	< .001	.335	.15	.23 to .44	< .001
MVPA overall	.249	.07	.11 to .39	.001	.242	.14	.11 to .38	.001
MVPA weekdays	.134	.04	.01 to .26	.039	.128	.11	.02 to .24	.026
MVPA weekend days	.350	.12	.20 to .50	< .001	.337	.16	.19 to .48	< .001

BMI — Body Mass Index, CPM — counts per minute, MVPA — moderate-to-vigorous physical activity.

a Adjusted for female age, BMI, Employment, number of other children, house hold cars and IMD score.

**Table 4:** Logistic regression displaying odds ratios for females meeting the recommended MVPA, predicted by whether males PA guidelines. Based on UK participants — 2012/13.

	Unadjusted			Adjusted^a^		
	OR	95% CI	P	OR	95% CI^a^	P
Overall	1.39	0.62 to 3.09	.421	1.42	0.66 to 3.08	.371
On weekdays	1.34	0.57 to 3.14	.506	1.58	0.59 to 4.25	.363
On weekend days	1.91	1.15 to 3.19	.013	1.92	1.13 to 3.27	.016

a Adjusted for female age, BMI, Employment, number of other children, house hold cars and IMD score.
